# Protocatechuic Acid, a Novel Active Substance against Avian Influenza Virus H9N2 Infection

**DOI:** 10.1371/journal.pone.0111004

**Published:** 2014-10-22

**Authors:** Changbo Ou, Ningning Shi, Qunhui Yang, Yu Zhang, Zongxue Wu, Baozhong Wang, Richard W. Compans, Cheng He

**Affiliations:** 1 College of Animal Science, Henan Institute of Science and Technology, Xinxiang, China; 2 Key Lab of Animal Epidemiology and Zoonosis, Ministry of Agriculture, College of Veterinary Medicine, China Agricultural University, Beijing, China; 3 College of Life Sciences, Agricultural University of Hebei, Baoding, China; 4 Department of Microbiology and Immunology, and Yerkes Vaccine Center, Emory University School of Medicine, Atlanta, Georgia, United States of America; China Agricultural University, China

## Abstract

Influenza virus H9N2 subtype has triggered co-infection with other infectious agents, resulting in huge economical losses in the poultry industry. Our current study aims to evaluate the antiviral activity of protocatechuic acid (PCA) against a virulent H9N2 strain in a mouse model. 120 BALB/c mice were divided into one control group, one untreated group, one 50 mg/kg amantadine hydrochloride-treated group and three PCA groups treated 12 hours post-inoculation with 40, 20 or 10 mg/kg PCA for 7 days. All the infected animals were inoculated intranasally with 0.2 ml of a A/Chicken/Hebei/4/2008(H9N2) inoculum. A significant body weight loss was found in the 20 mg/kg and 40 mg/kg PCA-treated and amantadine groups as compared to the control group. The 14 day survivals were 94.4%, 100% and 95% in the PCA-treated groups and 94.4% in the amantadine hydrochloride group, compared to less than 60% in the untreated group. Virus loads were less in the PCA-treated groups compared to the amantadine-treated or the untreated groups. Neutrophil cells in BALF were significantly decreased while IFN-γ, IL-2, TNF-α and IL-6 decreased significantly at days 7 in the PCA-treated groups compared to the untreated group. Furthermore, a significantly decreased CD4+/CD8+ ratio and an increased proportion of CD19 cells were observed in the PCA-treated groups and amantadine-treated group compared to the untreated group. Mice administered with PCA exhibited a higher survival rate and greater viral clearance associated with an inhibition of inflammatory cytokines and activation of CD8+ T cell subsets. PCA is a promising novel agent against bird flu infection in the poultry industry.

## Introduction

Avian influenza A (H9N2) virus (AIV) is transmitted sporadically from avian species to humans, causing mild diseases in immunosuppressed people. Eleven cases of human infection have been reported in Southern China since 1988 [1], [2]. Recently, a new strain of H7N9 virus attacked 132 people in eastern China and 33 have died. Local health officials believed people were contracting the H7N9 virus through direct contact with infected fowl. Hence, the avian-to-human interspecies transmission highlighted the potential for the emergence of a novel human pathogen [3]. Currently, avian influenza H9N2 subtype virus is still a major threat to the poultry industry in China due to only partial protection provided when using the available inactivated vaccines [4]. A culling strategy is not implemented in developing countries during outbreaks of avian influenza H9N2, and no practical measures have been confirmed to be effective in the control of the disease. Unfortunately the widespread application of two viral M2 proton channel blockers (amantadine hydrochloride and rimantadine) for the eradication of AIV in poultry has contributed to a high percentage of amantadine-resistant H5N1, H3N2 and H1N1 virus strains in the Chinese mainland [5], [6] and in India [7]. Therefore, amantadine and rimantadine are not recommended for prophylaxis or treatment of seasonal influenza infection in humans [6].

In poultry, antiviral and immunoadjuvant effects of several plants and/or their derivatives have been used to provide antiviral activity. Eugenia jambolana extracts had 100% virucidal activity against highly pathogenic avian influenza virus (HPAIV) H5N1 in tissue culture and in-ovo inoculated embryonated chicken eggs [8]. NAS preparation, a Chinese herbal medicine, prevented H9N2 virus-induced clinical signs in treated chickens, but transmission of the virus to untreated chickens was not interrupted [9]. Likewise, eucalyptus and peppermint essential oil preparations protected broilers against H9N2 virus infections [10]. Furthermore, statin/caffeine combination was confirmed to be as effective as oseltamivir in the reduction of HPAIV H5N1-induced lung damage and viral replication in mice [11]. Thus, the effectiveness of herbal and cytokines-based medications to protect against avian influenza virus should be seriously considered for further investigation.

In our previous study, protocatechuic acid (PCA) was found to improve the survival rate of chickens challenged with virulent infectious bursal disease virus [12]. Moreover, PCA is one of the organic acids with many bioactive properties, such as anti-inflammatory and anti-oxidative properties and the prevention of gastrointestinal carcinogenesis [13]–[15]. Such reports stimulated us to evaluate the treatment effectiveness and antiviral mechanism of PCA in an avian influenza virus mouse model.

## Materials and Methods

### Mice

SPF BALB/C female mice were purchased (Merial Experimental Animal, Beijing, China) and maintained in a negative pressure isolator. Mice weighing 18–22 g were selected and housed on food and water *ad libitum*. Animal studies were approved by the Animal Care and Use Committee of the China Agricultural University, Beijing, China. Clinically, a humane endpoint used in this study referred to the following signs in mice, such as a 20% weight loss over a few days, persistent decumbency and labored breathing. Investigators are obligated to make every effort to identify and humanely euthanize moribund animals. Once animals started to display clinical signs of illness, the animal were euthanized immediately according to the protocol of IACUC. All operations were performed to ameliorate animal suffering.

### Virus

A/Chicken/Hebei/4/2008(H9N2) virus was donated by Prof. Jian Qiao (China Agricultural University, Beijing) and propagated in 10-day-old embryonated SPF eggs. The virus stocks were collected and titrated as previously described [16]. The 50% mouse lethal dose (MLD_50_) was determined to be 10^6.25^ EID_50_/0.2 ml in allantoic fluid and this dose was administered intranasally.

### Animal studies

One-hundred-twenty female BALB/C mice were randomly divided into six groups (20 mice per group). One group was not infected, while the remaining 5 groups were challenged intranasally with 0.2 ml H9N2 virus dilution prior to treatment with drugs. One group of infected animals was used as an untreated group. Other groups received 0.2 ml of either PCA (Sigma-Aldrich, MO, USA) or amantadine hydrochloride (Sigma-Aldrich, MO, USA) twice by oral gavage post inoculation at 12 h intervals daily for a total of seven days. The 3 PCA doses used were 10, 20 and 40 mg/kg body weight, and the amantadine hydrochloride dose used was 50 mg/kg body weight dose [17]. The untreated group received equivalent amounts of physiological saline per mice while 20 mice were maintained as the control group with no infection and treatment. Clinical signs, body weight, and mortality were observed twice per day and the observation period lasted until the end of the experiment.

### Lung index and viral load

Three mice from each group were euthanized post-anesthesia by ether on day 7 and day 14 post infection. The body weights were measured and the lungs were aseptically removed and weighed. The lung index used here was the lung weight as a percentage of the body weight as previously described [18]. Fifty mg of sterile lung tissue from each mouse was then minced to prepare the supernatant from lung homogenates. Subsequently, 0.1 ml of serial dilutions from 10° to 10^7^ dosages was injected into the allantoic cavity of 10-day-old embryonated chicken eggs. Then these eggs were observed for 48 h. After that, hemagglutination titre of allantoic fluid from each egg was tested to determine the 50% egg infective dose (EID_50_).

### Assessment of lung inflammation

The numbers of leukocytes in bronchial alveolar lavage fluid (BALF) were assessed as previously described [16]. Briefly, three mice were euthanized on day 7 post infection and the lungs were lavaged twice with 1.0 ml sterile saline. Cell suspensions were centrifuged at 1000 rpm for 5 min and the cell pellets were re-suspended with 0.1 ml saline; then 5 µl cell suspensions were fixed on slides and stained with hematoxylin and eosin. The numbers of monocytes, neutrophils and lymphocytes in BALF were counted using light microscopy.

Post infection, five BALF samples from each group were collected on day 7 and day 14 and stored at −80°C until use. Subsequently the concentrations of IL-2, IL-6, IFN-γ, TNF-α and IL-10 in BALF were determined using a CBA mouse Th1/Th2/Th17 cytokines kit (BD Biosciences, Frankin Lake, NJ). The cytokines were determined by FACS Calibur and the results were analyzed using FCAP array.

### Phenotypes of peripheral blood lymphocyte

Peripheral bloods were collected from three mice in each group on day 7 and day 14 post-infection. Subsequently, 5 ml red blood cell lysis solutions was added into tubes and incubated for 30 min at room temperature and centrifuged at 1000 rpm for 5 min. Finally, cell pellets were re-suspended in 120 µl of a mixture of anti-mouse CD3-Percp, CD4-FITC, CD8-PE and CD19-APC (BD Biosciences, Franklin Lakes, NJ). The stained cells were analyzed using FACS Calibur as previously described [19].

### Statistical analysis

Statistical significance of the data was determined using SPSS 13.0 for Windows (SPSS Inc., Chicago, IL). The results are graphically expressed as means±SD with indicated significant differences between means.

## Results

### The effect of PCA on mouse survival following infection with influenza virus

**Figure 1:**
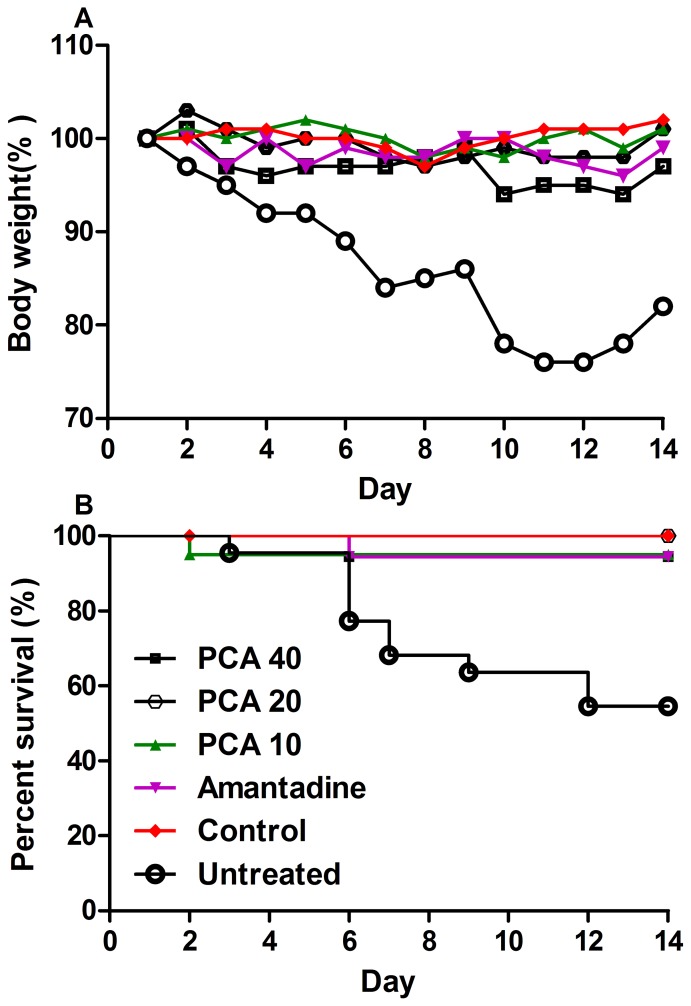
Therapeutic efficacy of protocatechuic acid (PCA) in mice infected with influenza virus A/Chicken/Hebei/4/2008(H9N2). (A) The assessment of mouse body weight post administration with PCA or amantadine hydrochloride as compared to the untreated or control animals; (B) The mice survival rate post administration with 10, 20 and 40 mg/kg of PCA or 50 mg/kg of amantadine hydrochloride twice daily by oral gavages at 12 h intervals for 7 days. The untreated group was inoculated with H9N2 virus without any medication.

Within three days post inoculation with avian influenza H9N2 virus mice exhibited a decreased food intake and decreased activity (data not shown). Body weight and mortality of all groups are shown in Fig. 1A and B. The body weight loss in the untreated group was significantly different from all other groups during the observation period. Mice treated with PCA exhibited a decreased body weight within the first week and began to increase their weight on day 8. The mean body weights of PCA-treated groups amounted to the level of the control group and were higher than that of the untreated group (Fig. 1A). Meanwhile, the survival was 94.4%, 100.0% and 95.0%, respectively in the 40 mg/kg, 20 mg/kg and 10.0 mg/kg PCA-treated groups, while the amantadine-treated group survival was 94.4%, while the survival in the untreated group was less than 60% by the end of the observation period (Fig. 1B).

### The effects of PCA on lung index and virus loads

**Figure 2:**
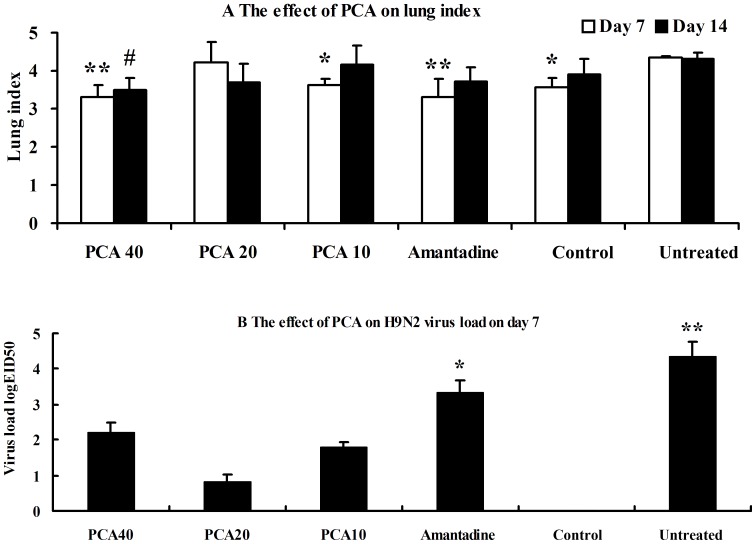
The effects of PCA on lung index (A) and virus load post infection with influenza virus A/Chicken/Hebei/4/2008(H9N2) (B). A.*^ #^ Indicates a statistically significant differences (P<0.05) in lung index between the untreated group and all other groups on day 7 (*) or day 14 (^#^). ** Indicates a statistically significant differences (P<0.01) in lung index between the untreated group and all other groups on day 7 (**). B. * Indicates a statistically significant difference (P<0.05) in virus load when the amantadine-treated group was compared with PCA-treated group on day 7. ** Indicates a statistically significant difference (P<0.01) in virus load when the untreated group was compared with the PCA- or amantadine-treated groups on day 7.

As shown in Fig. 2A, the mean lung index was increased post infection and was greater in the untreated group than in any of the other groups on either day 7 or 14. The lung indices of the 40 mg/kg group and the amantadine-treated group were not significantly different from each other (P>0.05) but were significantly lower than that of the untreated group (P<0.01), approaching the values of the control group on day 7. By day 7 the virus loads were less than 10^2.2^EID_50_/0.1 ml in the PCA-treated groups while no virus was detected in the control group (Fig. 2B). There was a significantly greater virus clearance in the three PCA-treated groups when compared to the amantadine group (P<0.05) or in the untreated group (P<0.01).

### The effects of PCA on lung inflammation

**Figure 3:**
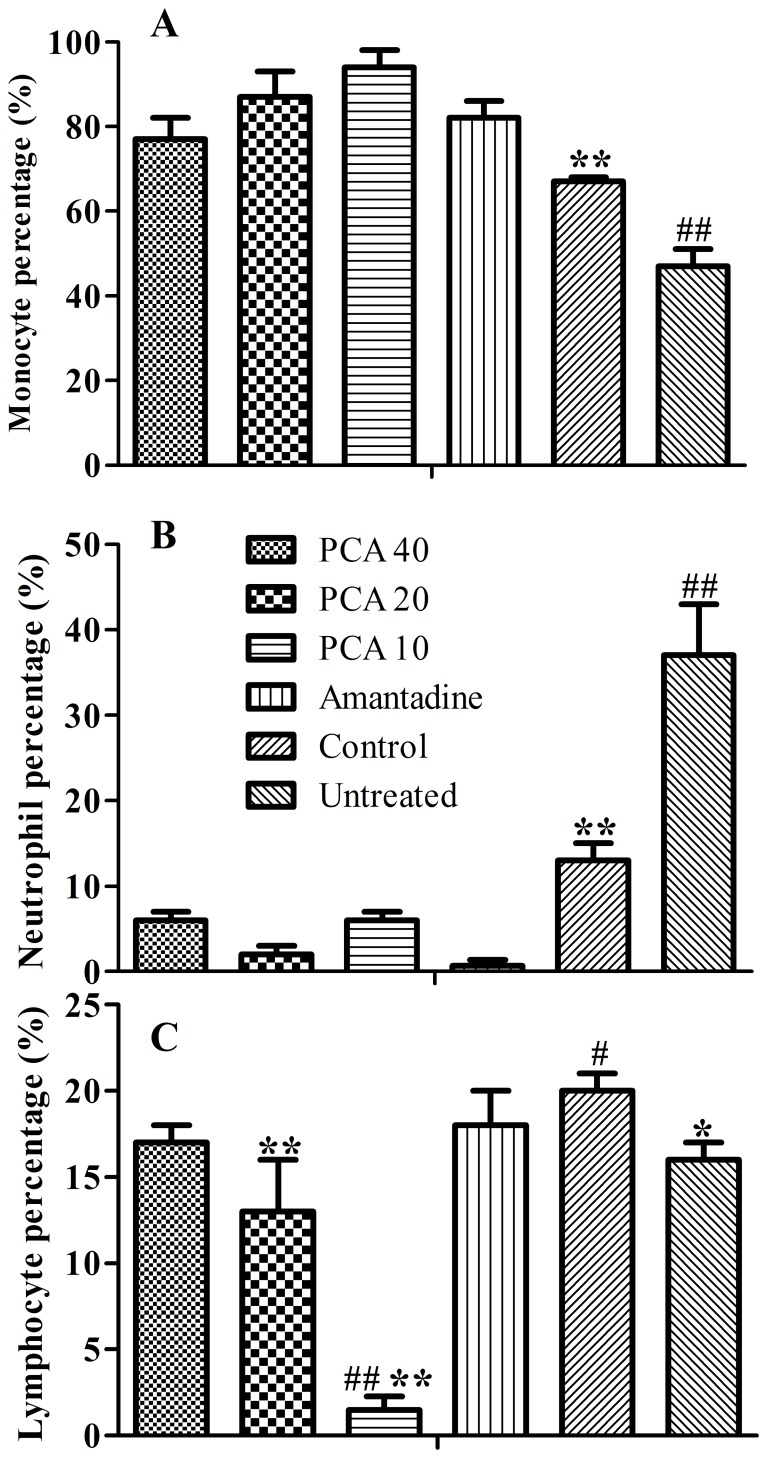
The effect of PCA on the percentage of monocytes (A), neutrophils (B) and lymphocytes (C) in bronchial alveolar fluid (BALF) on day 7 following infection. A. ^##^ Indicates a statistically significant difference (P<0.01) in monocytes percentage when the untreated group was compared with the PCA- or amantadine-treated groups on day 7. ** Indicates a statistically significant difference (P<0.01) in monocytes percentage when the control group was compared with the virus-treated groups on day 7. B. ^##^ Indicates a statistically significant difference (P<0.01) in neutrophils percentage when the untreated control group was compared with the PCA- or amantadine-treated groups on day 7. ** Indicates a statistically significant difference (P<0.01) in neutrophils percentage when the control group was compared with the virus-treated groups on day 7. C. ^##^ Indicates a statistically significant difference (P<0.01) in lymphocytes percentage when the untreated group was compared with 10 mg/kg PCA-treated group on day 7. ** Indicates a statistically significant difference (P<0.01) in lymphocytes percentage when the control group was compared with the 10 or 20 mg/kg PCA-treated groups on day 7. *or^#^ Indicates a statistically significant difference (P<0.05) in lymphocytes or neutrophils percentage when the untreated group was compared with the control group on day 7.

As shown in Fig. 3A, the proportions of monocytes in PCA- or amantadine-treated groups were significantly higher than those of the untreated and control groups (P<0.01) while neutrophil percentages in PCA- or amantadine-treated groups largely decreased on day 7 (Fig. 3B). Only lymphocyte percentage of the control group was a little higher (P<0.05) while the 10 mg/kg PCA group was significantly (P<0.01) lower than that of the untreated group. Meanwhile, the 20 mg/kg PCA group was also largely lower than that of the control group in the lymphocyte percentage in BALF (Fig. 3C).

### The effects of PCA on the cytokine levels in BALF

**Figure 4:**
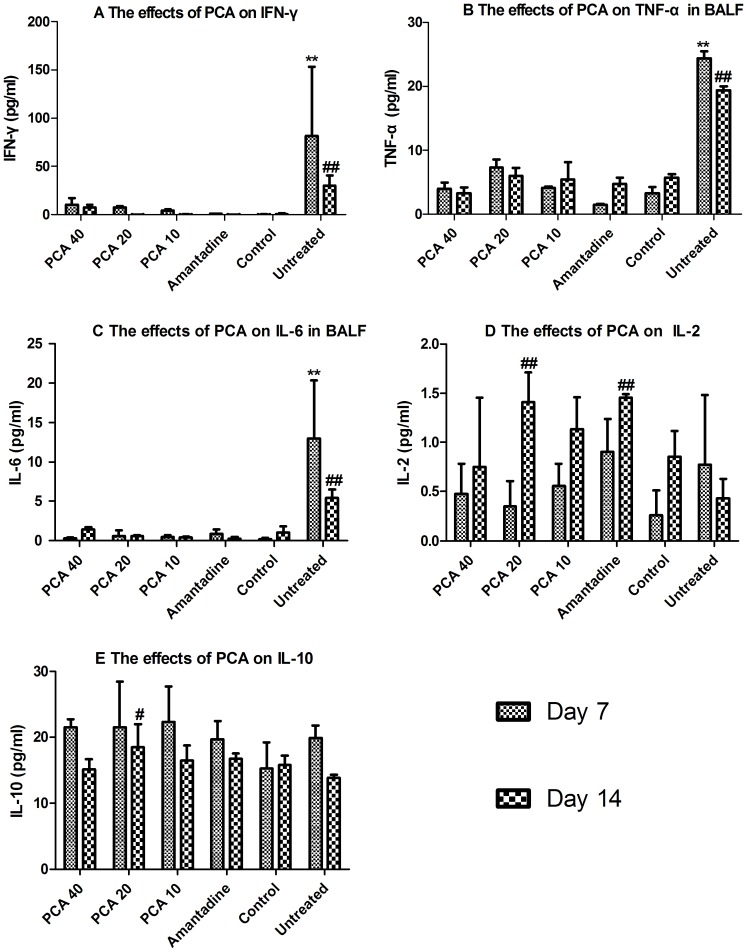
The effect of PCA on the levels of the inflammatory cytokines and antiviral cytokines (TNF-α, IFN-γ, IL-2, and IL-6) and IL-10 in BALF by ELISA assay on days 7 and 14 post infection. A. **^ ##^ Indicates a statistically significant difference (P<0.01) in the IFN-γ concentration when the untreated group samples were compared with all other groups on day 7 (**) or day 14 (^##^). B. **^ ##^ Indicates a statistically significant difference (P<0.01) in the TNF-α concentration when the untreated group samples were compared with all other groups on day 7 (**) or day 14 (^##^). C. **^ ##^ Indicates a statistically significant difference (P<0.01) in the IL-6 concentration when the untreated group samples were compared with all other groups on day 7 (**) or day 14 (^##^). D.^ ##^ Indicates a statistically significant difference (P<0.01) in the IL-2 concentration when the untreated group samples were compared with the 20 mg/kg PCA-treated group and amantadine-treated group on day 14. E.^ #^ Indicates a statistically significant difference (P<0.05) in the IL-10 concentration when the untreated group samples were compared with 20 mg/kg PCA-treated group on day 14.

Low levels of secretion of the inflammatory cytokines, IFN-γ, TNF-α and IL-6, were observed in the control group and in all drug-treated groups when compared with the untreated group on day 7 or 14 (P<0.01) (Fig. 4A–C). No significant difference was found between the PCA-treated groups and the amantadine group. In the case of IL-6 secretion, the mean values for all drug-treated groups were reduced to the levels seen in the healthy animals (Fig. 4C). By day 7 post-infection the mean IL-2 levels had increased in the all virus-infected groups compared with the control group, while on day 14 the mean IL-2 levels in the 20 mg/kg PCA- and amantadine-treated groups were significantly higher than that of the untreated group (P<0.01). Finally, there were no significant differences in the mean IL-10 concentration among the various groups, except in the case of the PCA20 group IL-10 mean value which was significantly greater than the untreated group and control group on day 14 (Fig. 4E).

### The effect of PCA on T and B cells in peripheral blood

**Figure 5:**
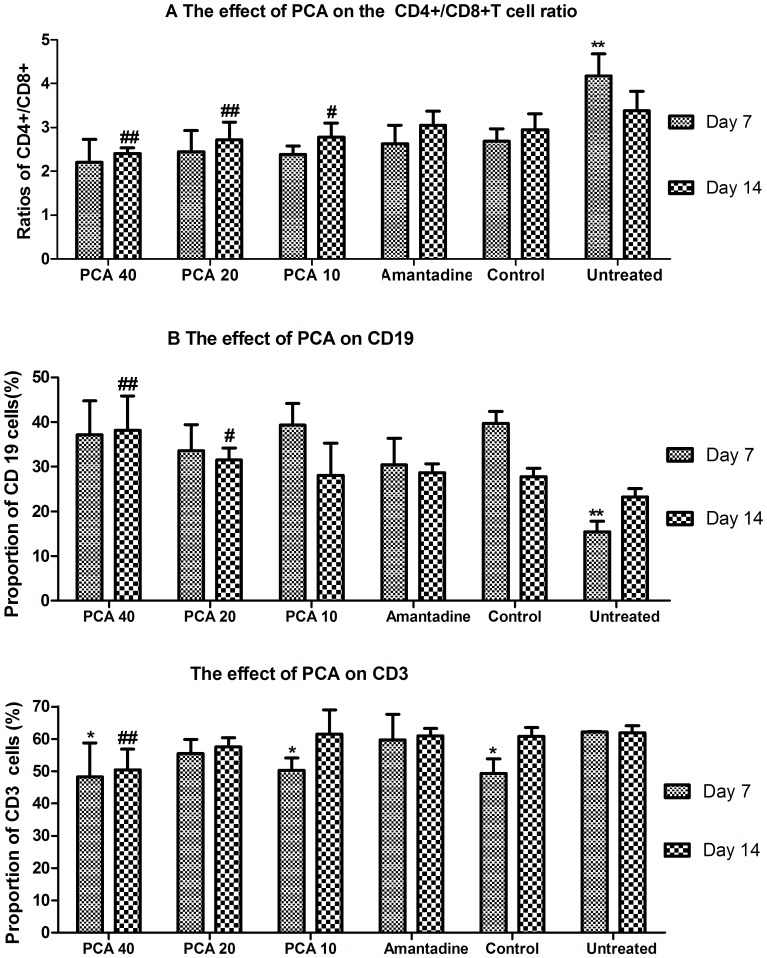
The effect of PCA on T cell subsets and B cell in peripheral blood cells on days 7 and 14 of mice infected with influenza virus. A. ** Indicates a statistically significant difference (P<0.01) in the ratio of CD4+/CD8+ cells between the untreated group and all other groups on day 7, and a significant difference between PCA treated groups and the untreated group on day 14 (##, P<0.01; #, P<0.05). B. ** Indicates a statistically significant difference (P<0.01) in the proportions of CD19+ B cell subset between the untreated group and all other groups on day 7. The proportion of CD19+ B cell subset between PCA-treated groups and the untreated group on day 14 (##, P<0.01; #, P<0.05). C. *Indicates a statistical difference (P<0.05) in the proportions of CD3+ T cells between the treated or healthy groups and the untreated group on day 7. ##Indicates a statistically significant difference (P<0.01) in the proportions of CD3+ T cells when the 40 mg/kg PCA-treated group were compared to the untreated group on day 14.

The CD4+/CD8+ ratios of the untreated group were significantly greater than those of the corresponding control groups and all drug-treated groups on day 7 (P<0.01). On day 14, the ratio of CD4+/CD8+ decreased in the untreated group, but was still significantly higher compared to the 40 and 20 mg/kg PCA-treated groups (P<0.01) (Fig. 5A).

Mean CD19 values were significantly less in the untreated group compared to the control group or any of the drug-treated groups on day 7 (P<0.01). However, no significant difference was found between the PCA groups and the amantadine group (Fig. 5B). The PCA 40 and PCA20 group proportion of CD19 cells remained significantly higher that that of the untreated group on day 14.

The CD3 cell concentrations in both the untreated group and amantadine-treated group were comparable while the PCA40 and PCA10 reduced to the level of the control group on day 7, and the percentage of CD3 cells in the 40 mg/kg group was significantly decreased compared with the untreated group on day 14 (Fig. 5C).

## Discussion

In the current study, following infection with avian H9N2 virus, mice receiving 10–20 mg/kg PCA had a higher survival rate, less lung damage, as well as higher virus clearance than the amantadine-treated group. Moreover PCA treatment resulted in reduced secretion of inflammatory cytokines in the lungs, while inflammatory cells were suppressed. These results strongly suggest that PCA have some anti-viral functions against avian influenza virus H9N2 in mouse model and future studies are needed to confirm the antiviral efficacy of PCA.

Drug-resistant virus variants occur very frequently, especially for influenza virus, while the development of antiviral agents is a long process requiring a huge financial investment [20], [21]. Since amantadine and osetamivir are prohibited in livestock and poultry applications, no commercial drugs are available for prophylaxis or treatment against avian influenza H9N2. Moreover, the current inactivated vaccines induce only partial protection during the seasonal prevalence, resulting in the frequent outbreaks in intensive chicken farms. New drugs are therefore urgently needed against avian influenza H9N2 virus.

In the present study, mice receiving 20 mg/kg PCA exhibited complete protection, while 94.4% protection was found in animals receiving a high dose of amantadine (Fig. 1B). The antiviral role of PCA in avian viral infection was also shown in a previous investigation in which chickens administered PCA were more than 85% protected post infection with infectious bursal disease virus (IBDV) [12], [22]. However, the mechanism of the PCA antiviral activity is unclear although there are several possibilities. First, PCA-induced immune regulation might improve the protection, particularly in the case of a virus infection. Our previous investigation showed that PCA contributed to the expansion of immune organs, e.g. spleen weight and lymph nodes both in mice and chicken models (data not shown). The PCA effects could also be due to metabolites of these phenolic polymers displaying biological activities that improve the survival rate of animals infected with virus in vivo. Previous findings confirmed that humate analogues, derived from protocatechuic acid not only improved immune responses in broilers [23], but also inhibited virus replication *in vitro* [24]. On the other hand, previous reports indicated that PCA did not result in inhibition of virus proliferation when added at the stage of viral adsorption or post adsorption [24], [25] *in vitro*.

Secondly, PCA may have contributed to the improvement of B cell function and increased antibody production against virus infection. PCA-treated mice displayed higher CD19 concentrations than the untreated group (Fig. 5B), suggesting improved antibody levels and effective eradication against virus infection. Furthermore, PCA may have reduced acute respiratory distress by down regulating inflammatory cytokines as well as the infiltration of inflammatory cells. Avian influenza virus is known to induce acute respiratory distress symptoms, which are characterized mainly by inflammatory cellular infiltration, interstitial and alveolar edema, and hemorrhage [26]. Several reports confirmed that high levels of IL-6 and TNF-α are associated with acute respiratory distress syndrome [26] and acute lung injury [27]–[29]. High levels of IFN-γ were also found in the lungs of mice infected with influenza virus accompanied by a peak of virus titer on day 6 post-infection [30]. In the present study, inflammatory cytokines such as IFN-γ, TNF-α, IL-2 and IL-6 were clearly suppressed by drug treatment while the anti-inflammatory cytokine IL-10 was up-regulated (Fig. 4). One previous report indicated that the effector T cells controlled lung inflammation during acute influenza virus infection by producing IL-10 [31]. Consequently, lung lesions, such as indicated by the lung index, and virus load were reduced. To some extent, acute respiratory distress induced by H9N2 was minimized, which probably contributed to the high survival in the PCA-treated groups. Our findings are consistent with previous reports that PCA pretreatment markedly reduced I/R-induced lung injury as indicated by histological alterations [32].

Post inoculation, influenza virus has been detected in the lung as early as one day after infection and subsequently replicates to extremely high levels [33], [34]. In the present study, we also found that virus loads in lungs taken from mice administered PCA were lower when compared with those of the untreated group or amantadine group, suggesting the efficient clearance of virus loads in the PCA-treated lungs. The virus eradication was associated with an increased proportion of monocytes, CD8+ subset T cells and a lower ratio of CD4+/CD8+ in the lungs. Recent reports confirmed that CD8+ cells provided protection against different strains of AIV by means of a large number of redundant mechanisms [35], [36]. High levels of virus-specific CD4+ T cells but not CD8+ cells predict severe pandemic influenza virus infection [37]. Interestingly, a higher proportion of CD8+ T cell subset might contribute to viral clearance as well as inhibiting inflammation in the mouse lungs post administration with PCA. Moreover, influenza A virus induces an immediate cytotoxic activity in all major subsets of peripheral mononuclear blood cells, i.e. CD3+, CD14+ and CD19+ cells [38]. Therefore, virus induced cytotoxicity could be partially blocked by PCA treatment. Previous studies also confirmed that PCA significantly reduced the number of total cells, neutrophils, and macrophages in the BALF induced by LPS administration [39]. However, further mechanistic investigations will be done to confirm the affected signaling pathways blocked by PCA during virus-induced activation.

In previous report, no toxicity was found in the rats administration with PCA at 1000 ppm or 100 mg/kg/day [40], [41] and our pilot study also confirm that LD_50_ is more than 5000 mg per kg in mice model, suggestion of non-toxic substance. More important, mice with 10–20 mg/kg of PCA are well protected as compared to the animals with 50 mg/kg of amantadine post inoculation with avian influenza virus H9N2. When mentioned to the restricted application of amantadine in livestock and poultry, it is urgent to find novel antiviral agents alternatives. PCA has been proved effective against H9N2 influenza virus infection in mice model, efficacy and compatibility against avian influenza virus infection should be done to verify application of PCA in chickens.
